# Attenuation Levels of Lung Fields in Quantitative Computed Tomography Evaluation Are Associated With Overall Survival and the Incidence of Acute Exacerbation in Combined Pulmonary Fibrosis and Emphysema: A Single‐Center Retrospective Observational Study

**DOI:** 10.1002/hsr2.73155

**Published:** 2026-09-01

**Authors:** Yusuke Suzuki, Yoshiaki Kitaguchi, Takumi Kinjo, Norihiko Goto, Masamichi Komatsu, Fumika Ueno, Yosuke Wada, Atsuhito Ushiki, Masanori Yasuo, Kiyoyasu Fukushima, Masayuki Hanaoka

**Affiliations:** ^1^ First Department of Internal Medicine Shinshu University School of Medicine Matsumoto Nagano Japan; ^2^ Departments of Clinical Laboratory Sciences Shinshu University School of Health Sciences Matsumoto Nagano Japan; ^3^ Department of Respiratory Medicine Japanese Red Cross Nagasaki Genbaku Isahaya Hospital Isahaya Nagasaki Japan

**Keywords:** acute exacerbation, combined pulmonary fibrosis and emphysema, density mask technique, overall survival, quantitative computed tomography

## Abstract

**Background and Aims:**

Combined pulmonary fibrosis and emphysema (CPFE) is characterized by the coexistence of emphysematous lesions and fibrotic interstitial lung disease. We assessed the relationships of quantitative computed tomography (CT)‐derived measures with clinical characteristics and outcomes in CPFE.

**Methods:**

At a single institution, 129 patients with CPFE were evaluated retrospectively. Baseline CT data were available for the entire cohort; 71 patients underwent repeat CT approximately 1 year later. The evaluated CT measures were low attenuation volume percentage (LAV%), high attenuation volume percentage (HAV%), diseased lung volume percentage (DLV%), erector spinae muscle cross‐sectional area (ESM_CSA_), and the main pulmonary artery‐to‐aorta diameter ratio. Associations of baseline values and annualized changes with overall survival and acute exacerbation incidence were examined.

**Results:**

None of the baseline quantitative CT measures was associated with the risk of death. In contrast, annualized changes in LAV%, HAV%, and DLV% were associated with the risk of death. Overall survival was shorter among patients with an annual DLV% increase of at least 1.5%. At baseline, HAV% and DLV% were associated with acute exacerbation incidence; in the longitudinal analysis, associations were observed for annualized changes in LAV%, HAV%, DLV%, and ESM_CSA_.

**Conclusion:**

In CPFE, annualized changes in LAV%, HAV%, and DLV% were associated with mortality and acute exacerbation incidence in their respective models. An annual DLV% increase of at least 1.5% was associated with shorter overall survival. Quantitative CT attenuation indices can inform risk assessment in CPFE.

## 1 Introduction

Combined pulmonary fibrosis and emphysema (CPFE) denotes fibrotic interstitial lung disease (ILD) with coexisting emphysematous lesions [1]. Software‐assisted computed tomography (CT) measurements have been used to quantify these concurrent emphysematous and fibrotic abnormalities.

CT‐based emphysema and ILD quantification can be performed with density masking, density histogram analysis, or texture classification [2]. Density masking uses predefined attenuation thresholds and is frequently applied.

Attenuation thresholds were first applied to quantify emphysema on CT with the density mask technique [3]. Subsequent image‐analysis approaches have included artificial intelligence methods based on convolutional neural networks [4]. Density mask‐based software remains clinically relevant: low attenuation volume percentage (LAV%), high attenuation volume percentage (HAV%), and diseased lung volume percentage (DLV%; LAV% + HAV%) have previously shown relationships with pulmonary function in chronic obstructive pulmonary disease (COPD), idiopathic pulmonary fibrosis (IPF), and CPFE [5].

In the same study, LAV% ≥ 1.5% and HAV% ≥ 12% were used as thresholds for emphysematous and fibrotic changes, respectively [5].

Quantitative CT also yields measures outside the lung parenchyma. Erector spinae muscle cross‐sectional area (ESM_CSA_), obtained from one axial CT image, has been linked to clinical outcomes in COPD, IPF, and pulmonary *Mycobacterium avium* complex disease [6, 7, 8, 9]. Pulmonary hypertension is a frequent complication of CPFE [10], and the main pulmonary artery‐to‐aorta diameter ratio (mPA/Ao ratio) has been studied as a CT indicator related to pulmonary hypertension and prognosis in IPF [11].

The relationships of density mask‐derived attenuation indices, ESM_CSA_, and the mPA/Ao ratio with clinical characteristics and outcomes in CPFE remain insufficiently described. We therefore evaluated these quantitative CT measures in relation to overall survival and acute exacerbation incidence.

## 2 Materials and Methods

### 2.1 Participants

The study cohort comprised 129 patients with CPFE whose first outpatient evaluation at Shinshu University School of Medicine occurred between April 2006 and March 2020. The report was prepared in accordance with the Strengthening the Reporting of Observational Studies in Epidemiology (STROBE) statement [12]. The collected clinical data included survival time and acute exacerbation incidence. Here, acute exacerbation referred to acute exacerbation of ILD rather than chronic obstructive pulmonary disease. The International Working Group criteria were applied [13]: respiratory deterioration developing acutely, typically within 1 month, together with new bilateral ground‐glass opacity and/or consolidation superimposed on fibrotic abnormalities on chest CT, when cardiac failure or fluid overload did not fully account for the deterioration. Patients were followed until death or study completion. The thresholds of LAV% ≥ 1.5% and HAV% ≥ 12% reported by Kitaguchi et al. [5] were used as operational attenuation‐based criteria indicating the presence of low‐ and high‐attenuation components, respectively, rather than as direct anatomical measurements of emphysema and fibrosis. Diagnosis of CPFE additionally required visual confirmation on CT of coexisting significant emphysema and significant pulmonary fibrosis [14, 15]. Two pulmonologists, Y.K. and Y.S., with 24 and 11 years of experience, respectively, evaluated the CT images without access to clinical data. All patients had a baseline smoking history of at least 10 pack‐years. Because of the retrospective design, smoking abstinence during follow‐up could not be systematically confirmed. Among the 129 patients, 71 underwent follow‐up CT at our hospital approximately 1 year after baseline. The cross‐sectional analyses related baseline LAV%, HAV%, DLV%, ESMCSA, and mPA/Ao ratio to overall survival and acute exacerbation incidence. The longitudinal analyses examined annualized changes in the same measures in relation to these outcomes.

This study was approved by the Institutional Research Ethics Committee of the Shinshu University School of Medicine (approval number: 5798; date of approval: March 28, 2023). The requirement for written informed consent was waived by the ethics committee because of the retrospective study design. Instead, the opt‐out document for this study was posted on the website of our department. After data collection, the data were processed anonymously to prevent access to information that could identify individual participants. The last date on which we accessed the data for research purposes was December 4, 2023. The retrospective observational study protocols were conducted according to the principles outlined in the Declaration of Helsinki of the World Medical Association.

### 2.2 Quantitative CT Assessment

Scanning was performed in the supine position during a full‐inspiratory breath hold with a 64‐row multidetector CT system (LightSpeed VCT; GE Healthcare, Buckinghamshire, UK). The protocol used 64 × 0.625 mm collimation, 120‐kV tube voltage, variable tube current, and a 0.4 s rotation time. Images of the lungs were reconstructed at 1.25 mm thickness with a standard algorithm. Whole‐lung datasets were analyzed with INTAGE Station LungVision version 3.0 (Cybernet, Inc, Tokyo, Japan), following previous reports [5, 16]. For voxels within the extracted lung field, LAV% was the proportion at ≤−950 HU and HAV% the proportion at ≥−750 HU; DLV% was calculated as LAV% + HAV%.

Using this software, density masking assigned voxels from the extracted lung fields according to prespecified CT attenuation thresholds. Central airway structures, including the trachea and bronchi, are excluded from the extracted lung fields; low‐attenuation airway lumens, therefore, did not contribute to LAV%. The method does not anatomically separate emphysema, fibrosis, or pulmonary vessels, and HAV% consequently includes vessels. The software did not permit calculation of HAV% after exclusion of pulmonary vessels. Accordingly, LAV%, HAV%, and DLV% were treated as attenuation‐defined lung‐field measures rather than direct measurements of pure emphysematous or fibrotic lesion volume.

ESM_CSA_ [6, 7, 8, 9] and the mPA/Ao ratio [11] were quantified with image‐analysis software (EV Insite; PSP, Co., Tokyo, Japan) according to previously published methods.

### 2.3 Pulmonary Function Tests

All patients underwent pulmonary function testing comprising spirometry and measurement of diffusing capacity of the lung for carbon monoxide (DLco), total lung capacity (TLC), residual volume (RV), and functional residual capacity (FRC) using a Chestac‐8900 system (CHEST Co., Ltd., Tokyo, Japan). The procedures followed earlier reports [5, 14, 15, 16, 17, 18] and were performed by two trained technicians in accordance with American Thoracic Society criteria.

### 2.4 Statistical Analyses

Continuous data are reported as mean ± standard deviation, whereas categorical data are reported as counts. All analyses were exploratory. Associations between quantitative CT measures and the risk of death were estimated using multivariable Cox proportional hazards models [19]. For acute exacerbation, multivariable Fine**–**Gray proportional subdistribution hazards models were fitted with death specified as the competing event [20]; their estimates are reported as subdistribution hazard ratios with 95% confidence intervals. Pairwise Pearson correlations among candidate variables were inspected for multicollinearity. Because DLV% is defined exactly as LAV% + HAV%, simultaneous inclusion of LAV%, HAV%, and DLV% would result in exact linear dependence. We therefore fitted Model 1 with LAV% and HAV% and Model 2 with LAV% and DLV%; the analogous annual‐change variables were used in the longitudinal models. Model 2 was treated as an alternative parameterization of Model 1 for presenting the composite attenuation‐defined measure and was not intended to assess incremental information beyond Model 1 or the component variables. Baseline and 1‐year CT measurements were compared with the Wilcoxon signed‐rank test. Kaplan**–**Meier estimates were used to describe overall survival, and groups were compared by the log‐rank test. All tests were two‐sided, and *p* < 0.05 was considered statistically significant. Analyses were conducted in EZR version 1.61 (Saitama Medical Center, Jichi Medical University, Saitama, Japan), which provides a graphical interface to R version 4.2.2 (The R Foundation for Statistical Computing, Vienna, Austria) [21].

## 3 Results

### 3.1 Baseline Characteristics

Baseline characteristics of the CPFE cohort are presented in Table 1. Reduced DLco was consistent with coexisting emphysema and pulmonary fibrosis, whereas elevated serum KL‐6 levels were consistent with pulmonary fibrosis.

**Table 1:** Baseline characteristics of the CPFE cohort (*n* = 129).

Variables	CPFE (*n* = 129)
Age, years	72.16 ± 7.89
Sex, male/female	118/11
Body mass index, kg/m^2^	23.45 ± 3.51
Smoking history, pack‐years	48.53 ± 32.29
Comorbidity	
Lung cancer	47
Acute exacerbation	27
Quantitative CT parameters	
CT‐derived total lung volume, mL	4063.89 ± 1027.99
LAV%, %	4.85 ± 6.65
HAV%, %	25.06 ± 11.27
DLV%, %	29.91 ± 11.04
ESM_CSA,_ cm^2^	34.15 ± 8.18
mPA/Ao ratio	0.81 ± 0.13
Pulmonary function tests	
VC, % predicted	85.99 ± 21.28
FVC, % predicted	88.30 ± 22.02
FEV_1_, % predicted	85.31 ± 19.20
FEV_1_/FVC, %	78.61 ± 11.55
FRC, % predicted	78.77 ± 21.75
RV, % predicted	96.50 ± 30.03
TLC, % predicted	88.77 ± 19.27
DLco, % predicted	45.54 ± 17.54
Laboratory data	
Serum LDH, IU/L	223.90 ± 64.51
Serum CRP, mg/dL	0.45 ± 0.84
Serum KL‐6, U/mL	898.71 ± 589.54

*Note:* Values are mean ± standard deviation (SD).

Abbreviations: CPFE, combined pulmonary fibrosis and emphysema; CRP, C‐reactive protein; CT, computed tomography; DLco, diffusing capacity of the lung for carbon monoxide; DLV%, percentage of diseased lung volume; ESM_CSA_, cross‐sectional area of the erector spinae muscles; FEV_1_, forced expiratory volume in 1 second;s; FRC, functional residual capacity; FVC, forced vital capacity; HAV%, percentage of high attenuation volume; KL‐6, Krebs von den Lungen 6; LAV%, percentage of low attenuation volume; LDH, lactate dehydrogenase; mPA/Ao ratio, ratio of diameter of main pulmonary artery to that of aorta; RV, residual volume; TLC, total lung capacity; VC, vital capacity.

### 3.2 Cross‐Sectional Study

None of the baseline quantitative CT measures was significantly related to the risk of death in the cross‐sectional Cox models (Table 2). In the Fine**–**Gray analyses of acute exacerbation, HAV% was significant in Model 1 and DLV% in Model 2 (Table 3), with death treated as the competing event.

**Table 2:** Multivariable Cox proportional hazards models of the risk of death in the cross‐sectional CPFE cohort (*n* = 129).

	Model 1			Model 2		
	HR	95% CI	*p* value	HR	95%CI	*p* value
LAV%	0.986	0.944–1.030	0.52	0.982	0.940–1.026	0.41
HAV%	1.004	0.977–1.032	0.76	—	—	—
DLV%	—	—	—	1.004	0.977–1.032	0.77
ESM_CSA_	0.981	0.946–1.018	0.31	0.981	0.946–1.018	0.31
mPA/Ao ratio	0.453	0.035–5.809	0.54	0.454	0.035–5.830	0.55

Abbreviations: CI, confidence interval; CPFE, combined pulmonary fibrosis and emphysema; DLV%, percentage of diseased lung volume; ESM_CSA_, cross‐sectional area of the erector spinae muscles; HAV%, percentage of high attenuation volume; HR, hazard ratio; LAV%, percentage of low attenuation volume; mPA/Ao ratio, ratio of diameter of main pulmonary artery to that of aorta.

**Table 3:** Multivariable Fine–Gray proportional subdistribution hazards models of acute exacerbation incidence in the cross‐sectional CPFE cohort (*n* = 129).

	Model 1			Model 2		
	sHR	95% CI	*p* value	sHR	95% CI	*p* value
LAV%	0.986	0.923**–**1.055	0.70	0.954	0.896**–**1.016	0.14
HAV%	1.034	1.007**–**1.063	0.02	—	—	—
DLV%	—	—	—	1.034	1.007**–**1.063	0.02
ESM_CSA_	0.999	0.942**–**1.059	0.97	0.999	0.942**–**1.059	0.97
mPA/Ao ratio	0.413	0.021**–**7.953	0.56	0.413	0.021**–**7.969	0.56

Abbreviations: CI, confidence interval; CPFE, combined pulmonary fibrosis and emphysema; DLV%, percentage of diseased lung volume; ESM_CSA_, cross‐sectional area of the erector spinae muscles; HAV%, percentage of high attenuation volume; LAV%, percentage of low attenuation volume; mPA/Ao ratio, ratio of diameter of main pulmonary artery to that of aorta; sHR, subdistribution hazard ratio.

### 3.3 Longitudinal Study

Table 4 compares the quantitative CT measures at baseline and the approximately 1‐year follow‐up. The mean interval was 1.1 ± 0.1 years. Significant within‐patient changes were observed in LAV%, HAV%, DLV%, and ESM_CSA_.

**Table 4:** Quantitative CT parameters at baseline and 1‐year follow‐up CT examinations.

	Initial CT	1‐year follow‐up CT	*p* value
LAV%, %	4.66 ± 6.34	5.71 ± 7.13	0.002
HAV%, %	24.58 ± 10.05	27.88 ± 14.82	0.008
DLV%, %	29.25 ± 10.44	33.60 ± 15.04	< 0.001
ESM_CSA_, cm^2^	33.31 ± 7.56	32.07 ± 8.16	0.03
mPA/Ao ratio	0.83 ± 0.14	0.85 ± 0.16	0.12

*Note:* Values are mean ± standard deviation (SD).

Abbreviations: CT, computed tomography; HAV%, percentage of high attenuation volume; DLV%, percentage of diseased lung volume; ESM_CSA_, cross‐sectional area of the erector spinae muscles; LAV%, percentage of low attenuation volume; mPA/Ao ratio, ratio of diameter of main pulmonary artery to that of aorta.

In the longitudinal Cox analysis, ΔLAV% and ΔHAV% were associated with the risk of death in Model 1, whereas ΔDLV% was associated with the risk of death in Model 2 (Table 5).

**Table 5:** Multivariable Cox proportional hazards models of the risk of death in the longitudinal CPFE cohort with follow‐up CT at approximately 1 year (*n* = 71).

	Model 1			Model 2		
	HR	95% CI	*p* value	HR	95% CI	*p* value
ΔLAV%	1.228	1.044–1.444	0.01	1.157	0.989–1.352	0.07
ΔHAV%	1.061	1.017–1.107	0.006	—	—	—
ΔDLV%	—	—	—	1.062	1.018–1.108	0.006
ΔESM_CSA_	1.017	0.932–1.109	0.71	1.018	0.933–1.110	0.69
ΔmPA/Ao ratio	6.618	0.270–162.20	0.25	6.410	0.264–155.40	0.25

Abbreviations: CI, confidence interval; CPFE, combined pulmonary fibrosis and emphysema; CT, computed tomography; ΔDLV%, annual change in percentage of diseased lung volume; ΔESM_CSA_, annual change in cross‐sectional area of the erector spinae muscles; HR, hazard ratio; ΔHAV%, annual change in percentage of high attenuation volume; ΔLAV%, annual change in percentage of low attenuation volume; ΔmPA/Ao ratio, annual change in ratio of diameter of main pulmonary artery to that of aorta.

The longitudinal Fine**–**Gray analysis of acute exacerbation identified ΔLAV%, ΔHAV%, and ΔESMCSA as significant in Model 1, and ΔDLV% and ΔESMCSA as significant in Model 2 (Table 6).

**Table 6:** Multivariable Fine–Gray proportional subdistribution hazards models of acute exacerbation incidence in the longitudinal CPFE cohort with 1‐year follow‐up CT obtained approximately 1 year after baseline (*n* = 71).

	Model 1			Model 2		
	sHR	95% CI	*p* value	sHR	95% CI	*p* value
ΔLAV%	1.238	1.057–1.451	0.008	1.129	0.965–1.319	0.13
ΔHAV%	1.097	1.040–1.157	0.001	—	—	—
ΔDLV%	—	—	—	1.097	1.040–1.157	0.001
ΔESM_CSA_	1.120	1.032–1.216	0.007	1.121	1.032–1.217	0.007
ΔmPA/Ao ratio	1.905	0.150–24.17	0.62	1.795	0.144–22.380	0.65

Abbreviations: CI, confidence interval; CT, computed tomography; CPFE, combined pulmonary fibrosis and emphysema; ΔDLV%, annual change in percentage of diseased lung volume; ΔESMCSA, annual change in cross‐sectional area of the erector spinae muscles; ΔHAV%, annual change in percentage of high attenuation volume; ΔLAV%, annual change in percentage of low attenuation volume; ΔmPA/Ao ratio, annual change in ratio of diameter of main pulmonary artery to that of aorta; sHR, subdistribution hazard ratio.

### 3.4 Kaplan–Meier Survival Analysis

**Figure 1:**
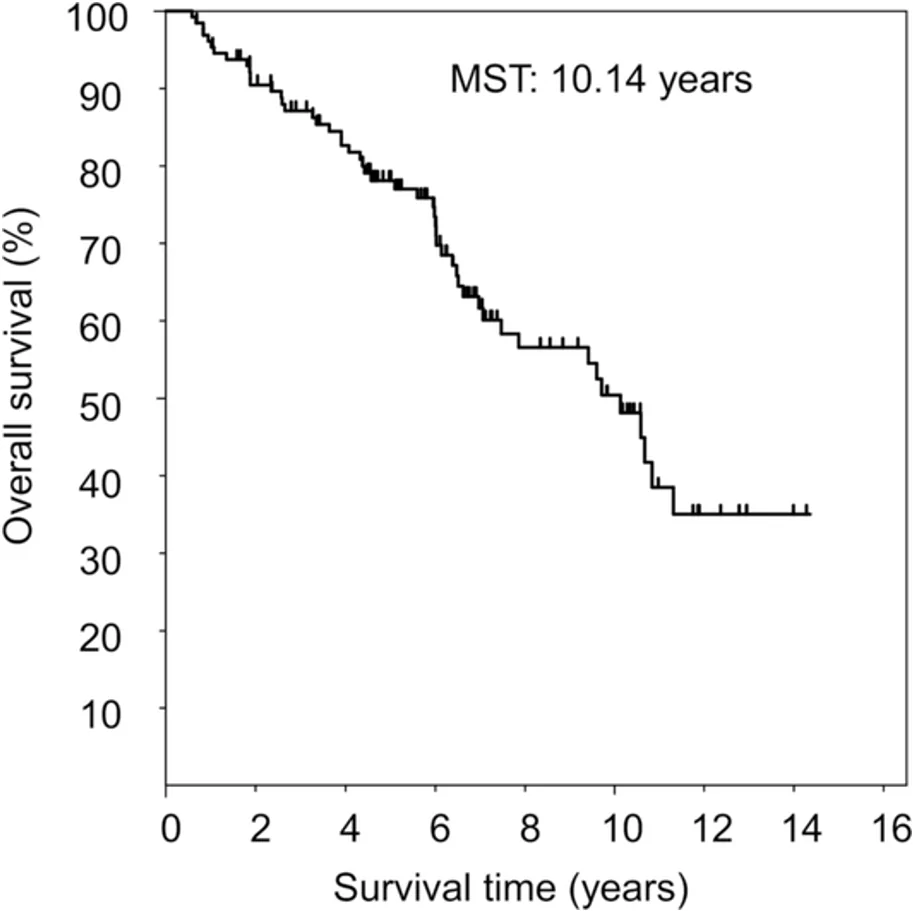
Kaplan–Meier curve showing overall survival in 129 patients with CPFE. CPFE, combined pulmonary fibrosis and emphysema; MST, median survival time.

**Figure 2:**
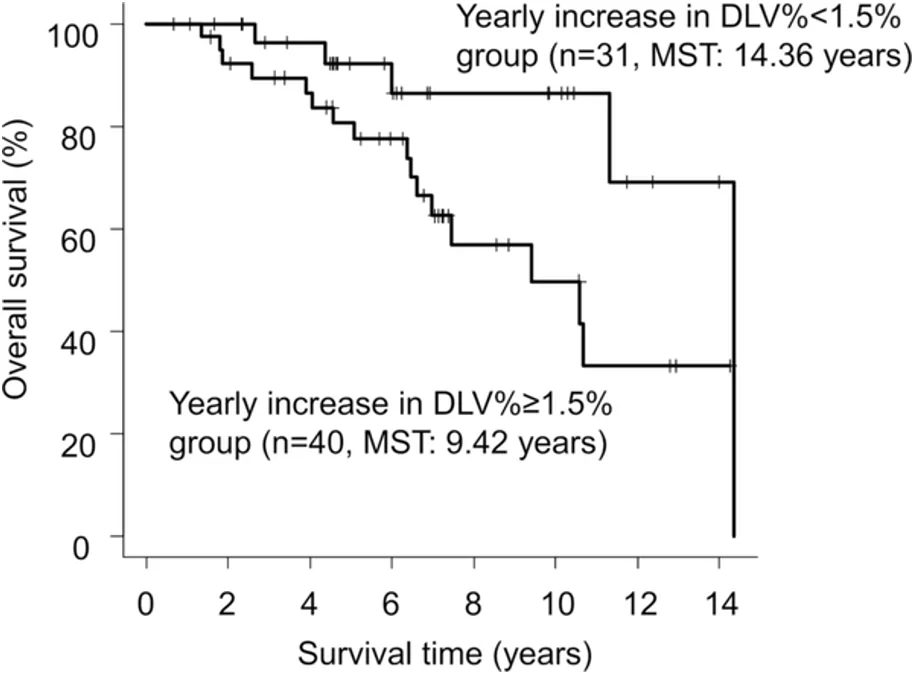
Kaplan–Meier curves comparing overall survival between patients with an annual DLV% increase < 1.5% (*n* = 31) and those with an annual DLV% increase ≥ 1.5% (*n* = 40). Differences in survival were assessed using the log‐rank test (*p* = 0.03). DLV%, percentage of diseased lung volume; MST, median survival time.

Figure 1 presents the Kaplan**–**Meier curve for the full 129‐patient cohort; median survival was 10.14 years. In the 71‐patient longitudinal cohort, receiver operating characteristic (ROC) analysis yielded an optimal Youden Index cutoff of 1.48% for annual DLV% increase to detect death at the median survival time (sensitivity, 80.8%; specificity, 63.2%; area under the ROC curve, 0.703). We rounded this value to 1.5%, defining groups with annual DLV% increase < 1.5% (*n* = 31) and ≥ 1.5% (*n* = 40). Overall survival was shorter in the ≥ 1.5% group (log‐rank *p* = 0.03; Figure 2). The median observation period was 5.76 years [0.68**–**14.36] for the cross‐sectional study and 6.12 years [0.59**–**14.36] for the longitudinal study.

## 4 Discussion

Baseline and longitudinal CT measures showed different associations with outcomes. Baseline attenuation indices were not significantly associated with the risk of death, whereas annualized increases in LAV%, HAV%, and DLV% were associated with this outcome. The inclusion of pulmonary vessels in HAV% may have introduced between‐patient variation unrelated to parenchymal lesions and may have attenuated cross‐sectional associations involving HAV% or DLV%. Calculating annual change may reduce a stable patient‐specific vascular contribution, but it does not guarantee removal of vascular effects or isolation of pure parenchymal disease progression because the vascular contribution may change between scans. Overall survival was also shorter among patients with an annual DLV% increase of at least 1.5%. Acute exacerbation incidence was associated with attenuation indices both at baseline and when assessed as annualized change. Thus, progression in attenuation‐defined abnormal lung volume may have clinical relevance in CPFE.

In CPFE, emphysema and fibrosis may coexist within the same lung region and form thick‐walled cystic lesions (TWCLs) on CT. TWCLs have been interpreted as emphysematous spaces distorted by surrounding fibrotic contraction and as radiologic‐pathologic features of CPFE involving bronchiolar lesions with features of both fibrosing interstitial pneumonia and emphysema [1, 22]. With the density mask technique, low‐attenuation areas may reflect not only emphysema but also TWCLs, honeycomb cysts, traction bronchiectasis, and mosaic attenuation related to small airway disease [1]. High‐attenuation areas may reflect the walls of TWCLs and honeycomb cysts as well as ground‐glass opacity and reticulation. Because these CPFE‐related structures may contribute to both LAV% and HAV%, DLV% may capture the combined attenuation‐defined abnormal lung burden. However, DLV% is mathematically determined by LAV% and HAV%. Accordingly, with LAV% included in both models, Model 2 (LAV% and DLV%) is an alternative parameterization of Model 1 (LAV% and HAV%) and does not add independent statistical information. We retained DLV% as a single composite summary of the proportions of lung voxels within either attenuation range, not to suggest that DLV% is superior to HAV%. This interpretation should be made cautiously because LAV%, HAV%, and DLV% are attenuation‐defined indices rather than anatomically segmented lesion volumes. The present study did not directly compare CPFE with COPD alone or ILD alone; therefore, differences in DLV% among these disease groups require further study.

Quantitative emphysema and ILD assessment on CT can use several methods. Texture analysis assigns lung regions to pathologic pattern categories, whereas artificial intelligence software can derive parenchymal lesion and airway‐volume measures [2, 4, 23, 24]. Although density masking has lower anatomical specificity, its explicit attenuation thresholds permit interpretation alongside visual CT assessment. Previous studies have linked DLV% to pulmonary function, including diffusing capacity of the lung for carbon monoxide, in patients with COPD and/or IPF [5, 25]. This previous evidence was one reason for evaluating DLV% in the present CPFE cohort and supports its physiological relevance as a composite attenuation‐defined index. However, it does not establish that DLV% is superior to LAV% or HAV%, and the same relationship was not directly examined in CPFE in those previous studies. DLV% may therefore represent the fraction of the lung within abnormal attenuation ranges, while 100 − DLV% represents the fraction outside the LAV% and HAV% ranges. The method in this study, however, did not anatomically separate vessels from emphysematous or fibrotic lesions. Consequently, our findings concern attenuation‐defined lung‐field indices and clinical outcomes; they do not show that exact anatomical lesion volumes were measured.

Although previous studies linked ESM_CSA_ to prognosis in COPD and IPF [7, 8, 9], no association with the risk of death was observed for ESM_CSA_ in either analysis of our CPFE cohort. ESM_CSA_ changed less over approximately 1 year than the attenuation indices, possibly contributing to this finding. Detection of clinically meaningful skeletal muscle loss on CT may require a longer interval. The mPA/Ao ratio was likewise not significantly associated with the risk of death, despite the frequent coexistence of pulmonary hypertension in CPFE and prior evidence linking this ratio to IPF prognosis [10, 11]. Pulmonary hypertension was not evaluated directly, and the mPA/Ao ratio did not change significantly between scans. Attenuation‐defined lung‐field measures may therefore change over shorter periods than ESM_CSA_ or the mPA/Ao ratio in CPFE.

In the longitudinal analysis of acute exacerbation, ΔLAV% was statistically significant in Model 1 but not in Model 2. Because ΔDLV% is defined as ΔLAV% + ΔHAV%, Model 2 is an algebraic reparameterization of Model 1. The change in the ΔLAV% coefficient, therefore, reflects a change in its interpretation and should not be taken as evidence that the low‐attenuation component is unrelated to acute exacerbation or that ΔDLV% is superior to ΔHAV%. Rather, ΔHAV% in Model 1 and ΔDLV% in Model 2 express the same fitted‐model information using component‐specific and composite parameterizations, respectively.

Several limitations should be considered. First, findings from this retrospective study at a single center using one CT scanner may not be generalizable to other settings. Second, comorbidities, particularly lung cancer and pulmonary hypertension, may have influenced prognosis. Lung cancer accounted for death in 17 of 129 patients in the cross‐sectional cohort and 4 of 71 patients in the longitudinal cohort. We did not exclude these patients because such comorbidities are common in CPFE and often occur during its clinical course. Third, treatment for COPD, IPF, or lung cancer may have affected outcomes. Even with this limitation, quantitative CT attenuation indices can provide information for risk assessment in CPFE. Fourth, CPFE was diagnosed by imaging criteria, including LAV% ≥ 1.5%, HAV% ≥ 12%, and visual confirmation of coexisting significant emphysema and significant pulmonary fibrosis; therefore, not all patients had pathological confirmation of ILD. Because CT has an established role in ILD diagnosis, including IPF, this approach was considered clinically acceptable, although future studies should examine whether associations differ among CPFE phenotypes defined by pathological diagnosis. Fifth, the longitudinal cohort included only 71 of 129 patients because some patients were referred elsewhere or did not undergo follow‐up CT at approximately 1 year. Baseline characteristics, including initial CT parameters, did not differ significantly between the full cross‐sectional cohort and the longitudinal cohort (Tables 1 and 4). Sixth, density mask analysis was based on CT attenuation thresholds and did not separately segment pulmonary vessels, emphysema, or fibrotic lesions. The software separates central airway structures, including the trachea and bronchi, from the extracted lung fields; therefore, LAV% did not include low‐attenuation areas within these airway lumens. However, HAV% included pulmonary vessels. The software could not generate a vessel‐excluded HAV% measurement. A method that separately segments pulmonary vessels might provide a more specific assessment of high‐attenuation parenchymal change and could yield different cross‐sectional or longitudinal associations. Such segmentation requires a more complex image‐analysis approach and was outside the scope of this study, which evaluated a simple attenuation‐threshold method. CT attenuation can also vary with inspiratory level, fluid status, and image noise. All quantitative CT scans were obtained during suspended full inspiration according to the institutional protocol, and all CT examinations were performed in the outpatient setting while patients were clinically stable enough for scheduled CT assessment. Large acute changes in total body fluid volume were therefore considered unlikely; however, smaller scan‐to‐scan differences in fluid status or pulmonary vascular volume could not be excluded, and the exact inspiratory level was not quantitatively standardized. Repeat‐scan reproducibility of LAV%, HAV%, and DLV% was not directly assessed. Longitudinal changes should therefore be interpreted as changes in attenuation‐defined indices, not exact changes in anatomical lesion volumes. Seventh, smoking abstinence during follow‐up was not systematically confirmed because of the retrospective design; the influence of smoking status during follow‐up on CT attenuation changes and outcomes could not be fully assessed.

## 5 Conclusions

In patients with CPFE, annualized changes in LAV%, HAV%, and DLV% were associated with mortality and acute exacerbation incidence in their respective models. An annual DLV% increase of at least 1.5% was associated with shorter overall survival. Quantitative CT attenuation indices can inform risk assessment in CPFE. However, DLV% is a composite measure of low‐ and high‐attenuation changes and does not provide information independent of its components.

## Author Contributions

**Yusuke Suzuki:** writing – original draft, data curation, formal analysis, investigation, methodology. **Yoshiaki Kitaguchi:** data curation, formal analysis, investigation, methodology, writing – review AND editing. **Takumi Kinjo:** data curation. **Norihiko Goto:** data curation. **Masamichi Komatsu:** data curation. **Fumika Ueno:** data curation. **Yosuke Wada:** data curation. **Atsuhito Ushiki:** data curation. **Masanori Yasuo:** data curation. **Kiyoyasu Fukushima:** data curation. **Masayuki Hanaoka:** data curation.

## Funding

This study was not supported by any sponsor or funder. No sponsor or funder had any role in the study design, data collection, data analysis, data interpretation, writing of the report, or the decision to submit the manuscript for publication.

## Ethics Statement

This study was approved by the Institutional Research Ethics Committee of the Shinshu University School of Medicine (approval number: 5798; date of approval: March 28, 2023).

## Consent

The requirement for written informed consent was waived by the Institutional Research Ethics Committee of the Shinshu University School of Medicine because of the retrospective study design. Instead, the opt‐out document for this study was posted on the website of our department. After data collection, the data were processed anonymously to prevent access to information that could identify individual participants.

## Conflicts of Interest

The authors declare no conflicts of interest.

## Transparency Statement

Dr. Yoshiaki Kitaguchi affirms that this manuscript is an honest, accurate, and transparent account of the study being reported; that no important aspects of the study have been omitted; and that any discrepancies from the study as planned have been explained.

## Data Availability

The data that support the findings of this study are available on request from the corresponding author. The data are not publicly available due to privacy or ethical restrictions. The datasets generated and/or analyzed during the current study are not publicly available because they contain clinical information that could compromise participant privacy. Deidentified data may be available from the corresponding author on reasonable request and with appropriate institutional approval. No custom analytic code or reusable study materials were generated.
